# Revisiting semicontinuous silver films as surface-enhanced Raman spectroscopy substrates

**DOI:** 10.3762/bjnano.10.105

**Published:** 2019-05-15

**Authors:** Malwina Liszewska, Bogusław Budner, Małgorzata Norek, Bartłomiej J Jankiewicz, Piotr Nyga

**Affiliations:** 1 Institute of Optoelectronics, Military University of Technology, 2 gen. Sylwestra Kaliskiego Street, 00–908 Warsaw, Poland; 2 Faculty of Advanced Technologies and Chemistry, Military University of Technology, 2 gen. Sylwestra Kaliskiego Street, 00–908 Warsaw, Poland

**Keywords:** metal island film, plasmon resonance, semicontinuous silver film, SERS, surface-enhanced Raman spectroscopy

## Abstract

Surface-enhanced Raman spectroscopy (SERS) is a very promising analytical technique for the detection and identification of trace amounts of analytes. Among the many substrates used in SERS of great interest are nanostructures fabricated using physical methods, such as semicontinuous metal films obtained via electron beam physical vapor deposition. In these studies, we investigate the influence of morphology of semicontinuous silver films on their SERS properties. The morphologies studied ranged from isolated particles through percolated films to almost continuous films. We found that films below the percolation threshold (transition from dielectric-like to metal-like) made of isolated silver structures provided the largest SERS enhancement of 4-aminothiophenol (4-ATP) analyte signals. The substrate closest to the percolation threshold has the SERS signal about four times lower than the highest signal sample.

## Introduction

Noble metal nanostructures exhibit exceptional optical properties. They can efficiently absorb and/or scatter visible and near infrared electromagnetic radiation [1]. The origin of the above phenomena lies in localized surface plasmon resonances (LSPR). LSPRs are light induced oscillations of free electrons in metallic nanostructures. The spectral position of an LSPR depends on the dielectric constant of the metal, surrounding dielectric, shape and size of the nanostructure, and its orientation with respect to the electric component of the electromagnetic field [1–2]. At resonance, the electric field near the surface of metallic nanostructures can be greatly enhanced and localized in nanoscale regions called “hot spots” [3]. These “hot spots” can be utilized in surface-enhanced Raman spectroscopy (SERS) [4], allowing for the detection of trace amounts of chemicals and biological materials, down to the single molecule or cell level [5].

SERS was discovered in the 1970s [6–8] and a considerable amount of research has been devoted to this topic. However, there is still a need for further development of reproducible and inexpensive SERS substrates [9]. SERS substrates can be fabricated by a multitude of techniques. These techniques can be divided into chemical and physical methods. Chemical methods allow for fabrication, in solution or on surfaces, of nanostructures of various shapes including: nanospheres [5], spheres coated with a thin dielectric shell [10], dielectric core-metallic shell particles [11–12], nanostars [13], microflowers [14] and aggregates [15], just to mention a few examples. Gold and silver nanostructured surfaces on substrates can be fabricated by self-assembly of colloidal particles into monolayers [16], in the form of island films by seeding with nanoparticles followed by a reduction of metal salt [17–18], or in the form of other high surface area structures [19–20]. Chemical fabrication methods are powerful in terms of the vast variety of attainable structure types, possible enhancement factors, and low cost of fabrication. However, reproducibility of SERS substrates can be an issue and the chemical compounds used for fabrication or stabilization of nanostructures can be a source of additional SERS signals [15], which may complicate SERS analysis with their use.

Various physical methods may be used to fabricate SERS substrates. Usually in these techniques silver or gold is deposited by physical vapor deposition (PVD) techniques. Nanostructures are obtained via various structuring methods. Electron beam lithography allows fabrication of planar [21] and 3D metallic structures [22]. Nanosphere lithography can be used to obtain nanotriangles [23] and nanocones [24]. Much attention has been also given to the deposition of metal onto nano- and micro-structured surfaces made of glass [25–26], GaN [27–29], Si [30], TiO_2_ [31], Al_2_O_3_ [32], Ti [33], polymers [34], or planar surfaces coated with nano/microspheres resulting in metal film on nanospheres MFON [35–36], and Au nanocrescents on a monolayer of polystyrene nanospheres [37]. Additionally glancing-angle deposition (GLAD) has been explored for the fabrication of vertical nanorods on planar substrates [38].

A special class of nanostructured surfaces are semicontinuous silver films (SSFs) [39–40] also known as metal island films [41], which are comprised of random fractal-type structures. SSFs can be fabricated on large area planar substrates using electron beam (or thermal) PVD techniques, and thus are simple to prepare and rather inexpensive. Island type structures can be also fabricated using pulsed laser deposition [42]. The SSFs form when 5–10 nm (mass thickness corresponding to hypothetical continuous film) of silver is e-beam deposited on a proper adhesion layer (for example silicon dioxide) [43–44]. The results of simulations and experimental studies show that the hot spots exist in SSFs [2,44–47], and hence they have been extensively studied as SERS substrates [40–41,48–55]. However, transmittance, reflectance and absorption are rarely reported in these studies, and it is difficult to link the optical properties of these nanostructures with their SERS performance. In our earlier initial study we investigated SSFs as SERS substrates [55], however the set of samples was limited to only four (with mass thickness from 3 nm to 10 nm) and they were characterized with a limited number of techniques.

The aim of this work is to revisit SSFs for their applications in SERS and perform a systematic study allowing for the correlation of the optical properties of silver film structures, with their SERS properties. We have investigated SERS enhancement of 4-aminothiophenol on nine SSFs with metal structures ranging from isolated particles, through percolated, to almost continuous film. We found that the largest SERS enhancement is observed for SSFs below the percolation threshold. The sample closest to the threshold has a SERS signal about four times lower than for the highest signal case.

## Experimental

### Materials

Silver deposition material (99.99%) and BK7 glass substrates were purchased from Umicore. For glass substrates cleaning process, we used sulfuric acid (97%; Fluka), hydrogen peroxide (30%; Chempur), ethanol (96%; Chempur) and deionized water. SERS analyte 4-aminothiophenol (97%) was purchased from Sigma-Aldrich.

### Fabrication of semicontinuous silver films

SSFs were fabricated on BK7 glass substrates using the electron beam PVD technique. The substrates were first cleaned with piranha solution (H_2_SO_4_/H_2_O_2_ 3:1) for 30 minutes, then rinsed with deionized water, followed by multiple rinses with ethanol. The substrates were then placed in an electron beam vacuum evaporation chamber. The base pressure of the chamber was about 2 × 10^−6^ mbar. Deposition was performed at room temperature. Glass substrates were first coated with 10 nm thick layer of silicon dioxide (SiO_2_). Next, without breaking vacuum, silver was deposited on the substrates. Two depositions were performed and in each of them several substrates were located at a different distance from the evaporation source to fabricate films with different thicknesses. In such way in two depositions a total of nine samples were fabricated. In order to ensure uniform thickness of SiO_2_, the substrates were rotated during the deposition process. The thickness of deposited films was monitored with quartz crystal microbalance.

### Characterization of semicontinuous silver films

Optical properties of SSFs were characterized using UV–vis–NIR Perkin Elmer Lambda 900 spectrometer. Transmittance was measured using a standard detector, while reflectance was measured with an integrating sphere module. Absorption was calculated assuming the sum of transmittance, reflectance, and absorption is 100%. The morphology of the fabricated structures was measured using a Quanta 3D FEG Dual Beam scanning electron microscope (SEM) and an atomic force microscope (AFM). The SEM images of SSFs were converted to black and white and metal coverage was calculated. The AFM maps were collected using an NTEGRA atomic force microscope from NT-MDT company. The surface topography measurements were made in semi-contact mode. We used HA_NC ETALON (NT-MDT) probe with 140 kHz ± 10% resonant frequency, force constant of 3.5 N/m ± 20% and standard tip curvature radius less than 10 nm. The thickness of SSFs was measured on the edge (step) formed through removing of a part of the silver film from the substrate (using a blade).

### SERS measurements

The 4-aminothiophenol was used as a SERS analyte. In order to form a monolayer of 4-ATP on the silver nanostructures we immersed the SSF substrates into 10 mM ethanol solution of 4-ATP for 1 hour. Longer incubation times did not result in SERS signal increase. The excess 4-ATP molecules were removed by rinsing the SSF samples with ethanol. The solvent was allowed to evaporate slowly.

The SERS measurements of 4-ATP analyte on SSFs samples were carried out using a Renishaw inVia Raman microscope. The Raman signal was acquired in the spectral range of 250–2000 cm^−1^ using laser radiation with a wavelength of 785 nm. The laser excitation power was 75 µW on the sample. The laser beam was directed to the sample through a 50× (N.A. = 0.75) objective lens. We used a 10 second integration time. On each SSF sample we measured the Raman signal in three locations and averaged. The wavelength of the instrument was calibrated using an internal silicon wafer, the spectrum was centered at 520.5 cm^−1^.

## Results and Discussion

**Figure 1:**
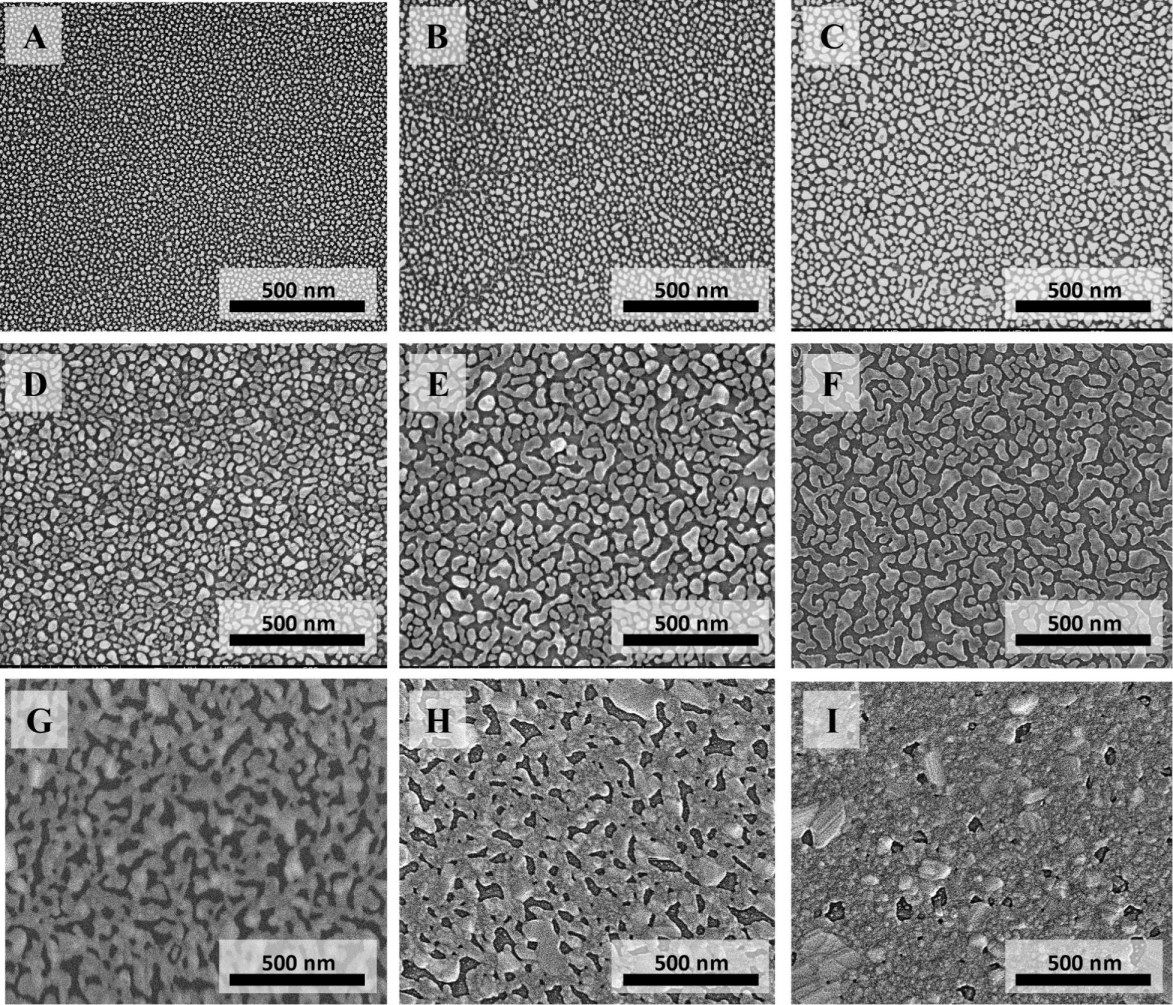
(A–I) SEM images of SSFs with different Ag thickness.

**Figure 2:**
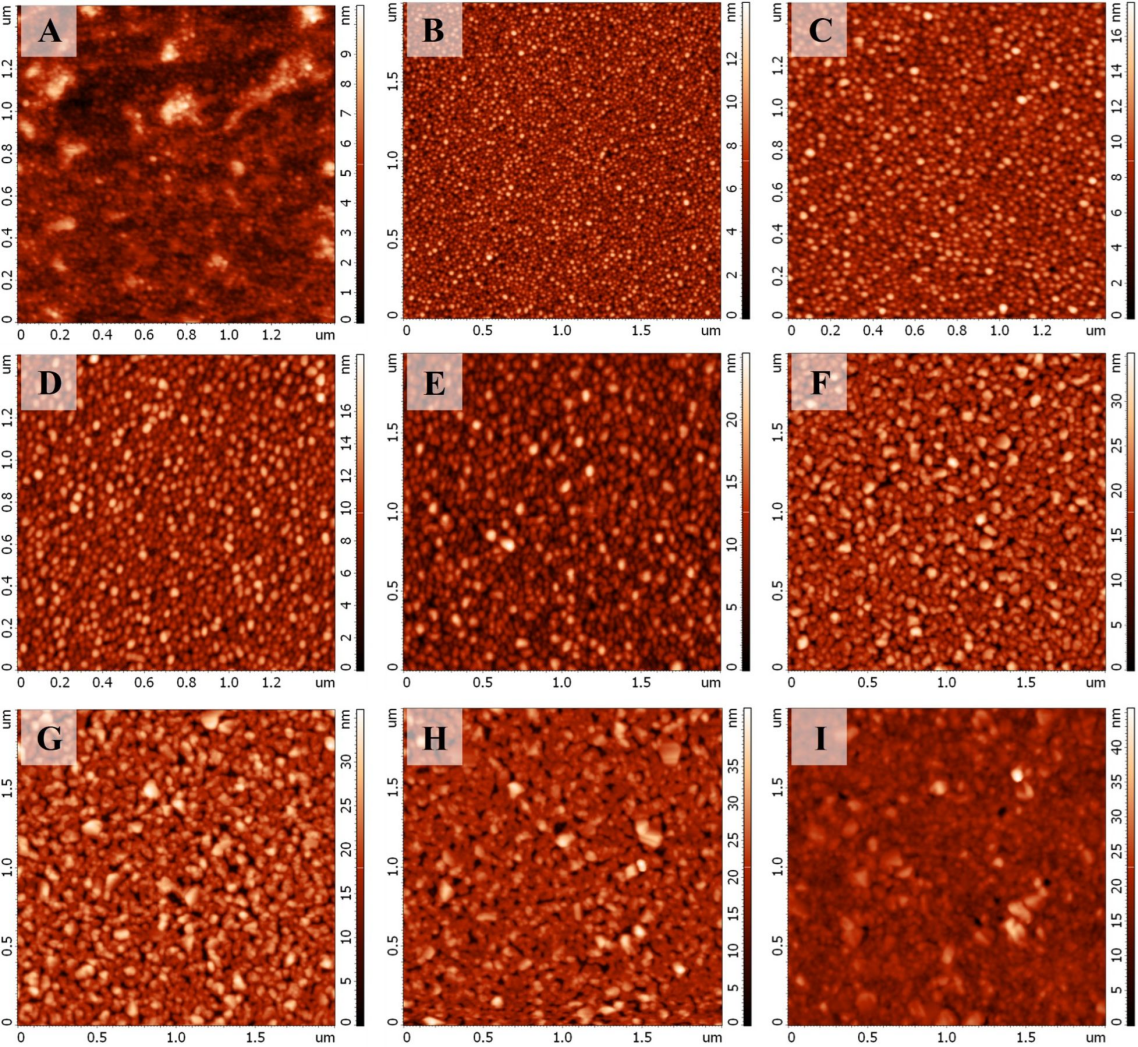
(A– I) AFM maps of SSFs with different Ag thickness. Samples A, C and D have a different scan area than the other samples.

We fabricated a series of nine (named A–I) SSF samples with different silver thickness (Figure 1 and Figure 2). The SSFs were prepared on glass substrates using electron-beam PVD (see Experimental section for details). We used transparent substrates in order to be able to measure transmittance and reflectance. We performed two depositions. Each deposition was concurrently performed on several substrates located at different distances from the evaporation source to get different silver film thicknesses. In such way in two depositions a total of nine SSF samples with different film thicknesses were fabricated. SEM images of the A–I SSFs are presented in Figure 1. They are arranged in a way that metal coverage (presented in Table 1) increases from A to I. Changes of the metal film thicknesses resulted in different SSFs morphologies. Sample A is comprised of small isolated particles. As the thickness increases the nanoparticles grow and their total number (per surface area) decreases (samples B–D). Next, the particles connect forming irregular fractal type shapes (samples E, F). As the amount of deposited metal further increases the metal coverage increases and the film reaches the percolation threshold – the transition from dielectric-like to metal-like (sample G is already above percolation threshold), where a metallic path forms across the sample. Finally, silver covers almost the entire surface of the substrate (sample I). The percolation point can be deducted from SEM images. One has to determine silver coverage or thickness for which a Ag path across the image/sample is formed. This can be performed through for example image analysis techniques. In the set of our nine samples we do not have a sample “at percolation”. The percolation would happen for a hypothetical sample between sample F and G (as for sample F we do not observe continuous silver path and for sample G several paths across the SEM image exist).

**Table 1:** The AFM-determined thicknesses of semicontinuous Ag films and estimated values of metal filling factors.

Sample	AFM-determined Ag thickness [nm]	Ag filling factor

A	8.75	0.428 ± 0.029
B	13.24	0.430 ± 0.022
C	13.37	0.513 ± 0.012
D	12.27	0.522 ± 0.001
E	20.46	0.549 ± 0.009
F	24.51	0.572 ± 0.008
G	23.88	0.641 ± 0.024
H	25.42	0.768 ± 0.021
I	22.35	0.937 ± 0.009

In order to determine the physical thickness of the SSFs we carried out AFM studies (data presented in Table 1). For each of the SSFs a part of the silver film was removed to form a step like structure with two distinct areas (glass with silicon dioxide and glass with silicon dioxide and silver film) of different height. A several micrometer square AFM scan (not presented) of such step-like structures provides an estimate of the SSF height but does not show the fine structure of the SSF. In order to visualize the height and morphology of silver nanostructures we performed AFM scans of relatively small areas of SSFs (Figure 2). The AFM data in Figure 2 corresponds well with the SEM images presented in Figure 1. The size of particles increases for samples from A to I. Also, the measured height range increases.

**Figure 3:**
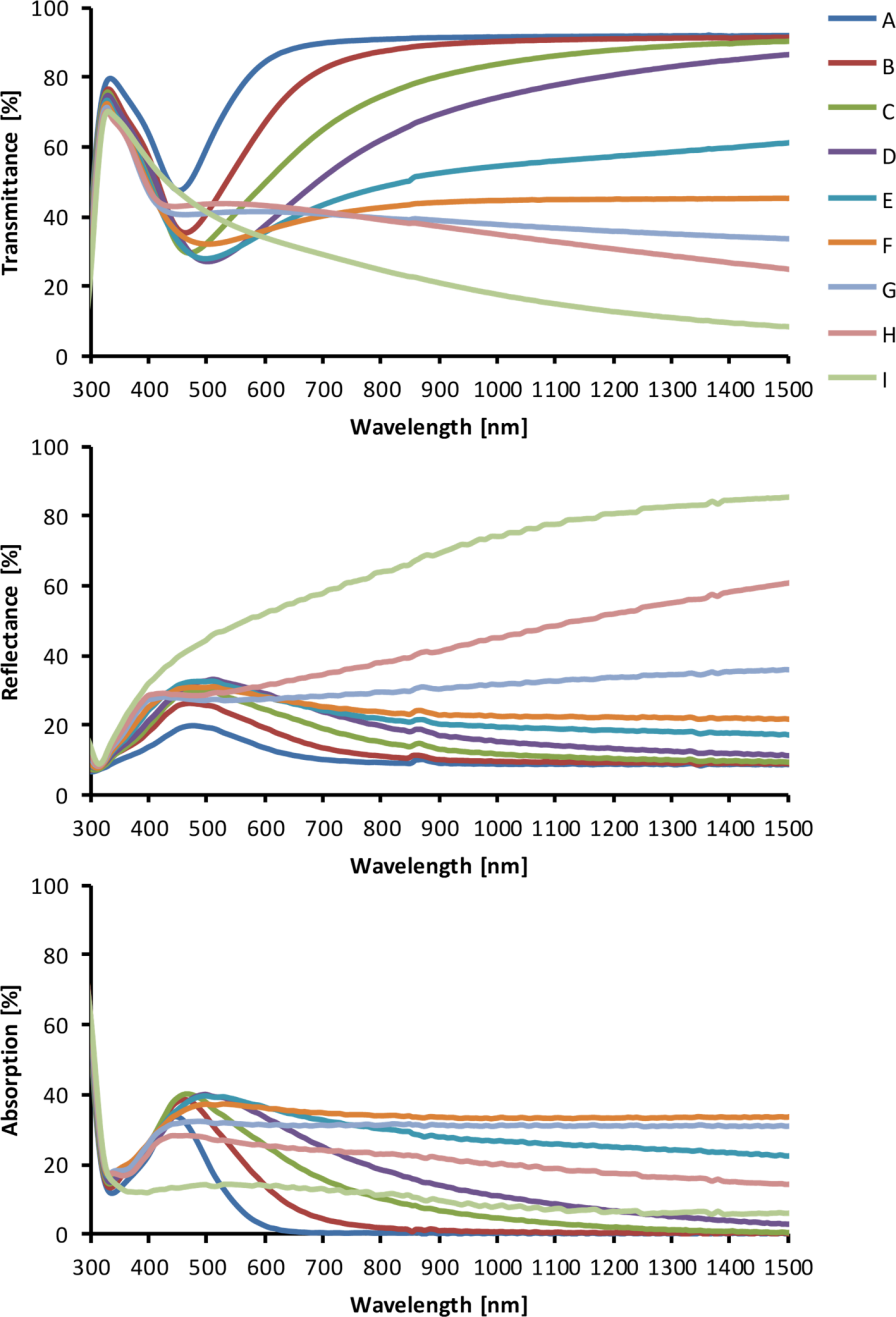
Transmittance, reflectance and absorption spectra of A– I SSF films.

The morphologies of SSFs strongly influence their optical properties. The measured transmittance, reflectance, and absorption of fabricated SSFs are presented in Figure 3. Film A has an absorption peak centered at about 435 nm. This peak corresponds to LSPR of isolated silver nanoparticles. With increased silver coverage the absorption peak broadens and shifts to longer wavelengths (samples B–E). Sample F has almost wavelength independent absorption (as well as transmittance and reflectance). Such behavior is known for metal films close to percolation [56]. This is because almost percolated films are comprised of nanostructures with different particle sizes and shapes, which results in absorption of electromagnetic energy across a broad wavelength range extending from the visible to the far infrared. For samples above percolation G-I reflectance increases (as the film is more metallic) and both absorption and transmittance decrease.

**Figure 4:**
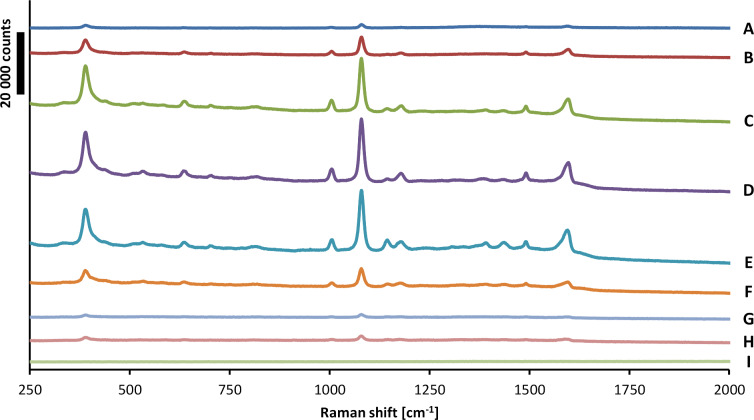
SERS spectra of 4-ATP molecules on A–I SSFs.

SERS properties of fabricated SSFs were examined using 4-ATP. We used 785 nm excitation wavelength. The 4-ATP SERS spectra measured on all SSFs are presented in Figure 4. In the spectra there are three prominent peaks at 389, 1080 and 1595 cm^−1^. The first two can be assigned to the C–S stretching mode and the third to C–C stretching mode. Four relatively weak peaks at 1180, 1392, 1437 and 1490 cm^−1^ can be assigned to the same C–H bending and a combination of C–C stretching and C–H bending. The characteristic peaks observed in 4-ATP spectra are in agreement with peaks reported in the literature [57].

**Figure 5:**
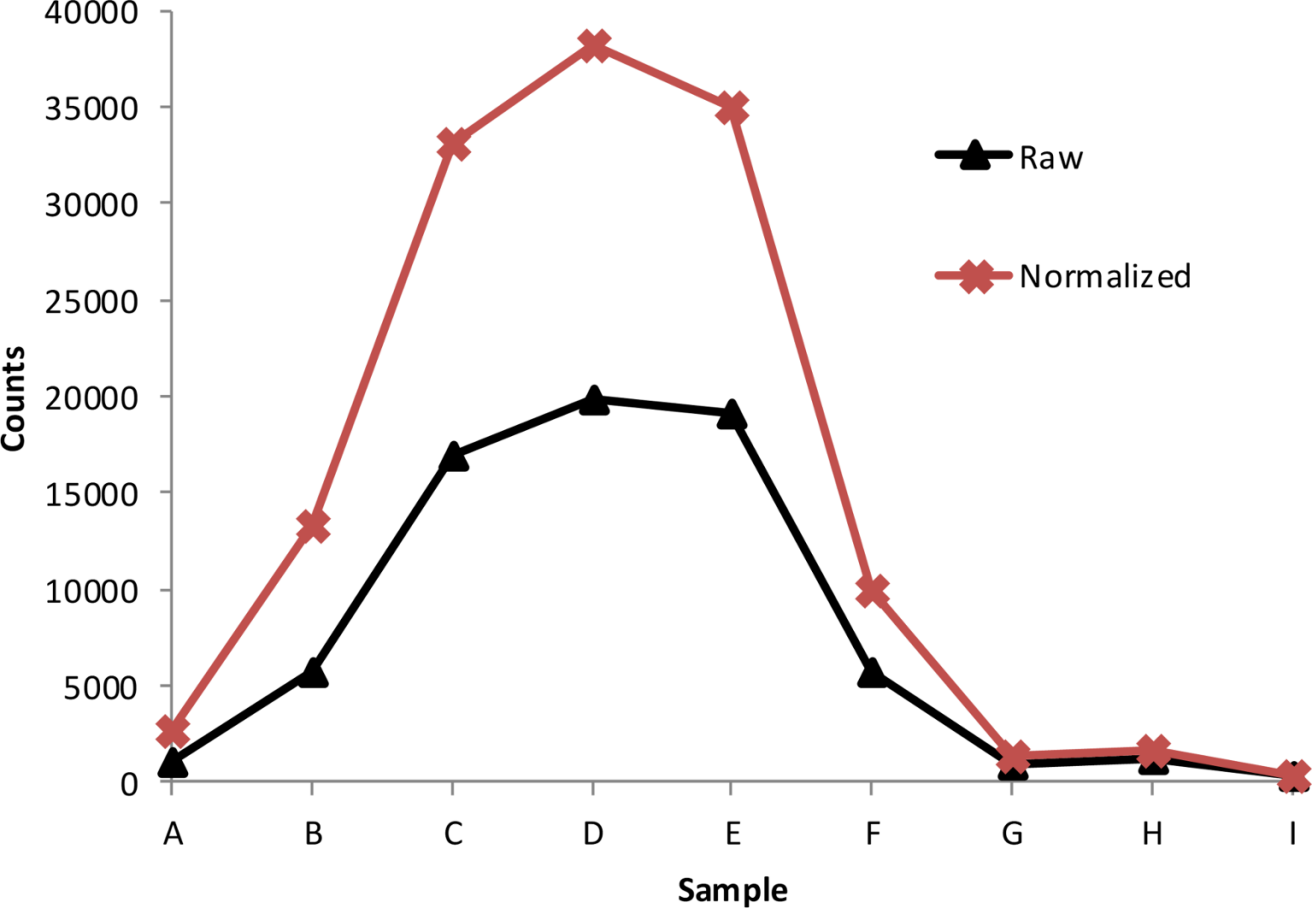
SERS signal of 1080 cm^−1^ band measured on SSFs A–I (Raw) and normalized by their metal coverage (Normalized).

At the excitation wavelength of 785 nm the SSFs have different absorption (Figure 3), from 0% for sample A, increasing to about 34% for sample F and decreasing to 11% for sample I (Figure 3). Despite low absorption at the excitation wavelength, a weak SERS signal was detected on sample A. This is in an agreement with previous reports of good quality SERS spectra obtained on substrates with a LSPR far away from the excitation laser wavelength [58]. We did not observe SERS signal for the sample I with the highest metal coverage. The highest SERS signal was obtained for samples C, D and E. These samples have different absorption at excitation wavelength, but they have similar morphology. These three samples are below percolation (SEM images in Figure 1) and have similar metal coverage (0.51–0.55; Table 1). Since each of the nine SSF samples has a different metal coverage there is a different surface area available for 4-ATP binding, thus different number of molecules per unit area. In order to exclude this effect, we normalized the measured SERS signals by the metal coverage (presented in Table 1). The measured and metal coverage corrected signals of the 1080 cm^−1^ peak for A–I SSFs are presented in Figure 5. For both measured and normalized case the samples C–E show high SERS signal with the D sample having the highest signal. The 4-ATP SERS signal recorded on SSFs above percolation (samples G and H) was at least an order of magnitude lower than that for the case of the three samples with highest SERS signal. For the case of SSFs fabricated using our protocol it is possible to determine if the film is below percolation from the transmittance and/or reflectance measurements (transmittance increases, reflectance decreases for wavelengths in the range of about one micrometer) without the need for expensive and time-consuming structural characterization. This could be used as a quick method for initial optimization of SSF thickness for high SERS signal.

Calculation of the SERS enhancement factor of a SERS substrate is extremely difficult since a proper reference sample is needed and there is an ongoing debate in the community regarding the appropriate procedures [59]. We decided to estimate the lower limit of the enhancement factor by adopting an approach similar to one used in the reference [20]. We compared the metal coverage normalized SERS signal of the D sample with the signal of the almost continuous I sample. As no SERS signal was observed on the sample I, we assumed that the upper limit of the signal is the peak-to-peak noise measurement [20]. By dividing the silver coverage corrected signal of the Raman band at 1080 cm^−1^ by the peak-to-peak noise of the measurement on the sample I we obtained a lower limit of the enhancement factor of about 630.

## Conclusion

We have fabricated semicontinuous silver films with various morphologies, ranging from isolated particles, through percolated to almost continuous film, and investigated their performance as SERS substrates. The SERS activity of studied substrates was explained with relation to their morphologies and optical properties. SERS tests using 4-ATP as an analyte confirmed that the Raman signal enhancement is strongly dependent on the morphology and optical properties of the substrate, and particularly absorption of the film. We found that films below the percolation threshold, composed of isolated silver structures, provide the highest SERS signal. For the sample closest to the percolation threshold the SERS signal is about four times lower than in the highest signal case. The semicontinuous silver films above percolation threshold produced 4-ATP SERS signal at least an order of magnitude lower than the best film.
